# Recent advancements in wound management: Tailoring superwettable bio-interfaces

**DOI:** 10.3389/fbioe.2022.1106267

**Published:** 2022-12-07

**Authors:** Dongsheng Zhong, Hongbo Zhang, Zhengxin Ma, Qiangwei Xin, Yongping Lu, Ping Shi, Meng Qin, Jianshu Li, Chunmei Ding

**Affiliations:** ^1^ Guangyuan Central Hospital, Guangyuan, China; ^2^ State Key Laboratory of Polymer Materials Engineering, College of Polymer Science and Engineering, Sichuan University, Chengdu, China

**Keywords:** superwettability, bio-interface, hemostasis, exudates, wound management

## Abstract

Skin tissue suffering from severe damages fail in self-regeneration. Proper wound dressings are highly demanded to protect the wound region and accelerate the healing process. Although large efforts have been devoted, there still exist disturbing dilemmas for traditional dressings. The exquisite design of bio-interface upon superwettable materials opens new avenues and addresses the problems perfectly. However, the advancements in this area have rarely been combed. In light of this, this minireview attempts to summarize recent strategies of superwettable bio-interfaces for wound care. Concentrating on the management of biofluids (blood and exudate), we described superwettable hemostatic bio-interfaces first, and then introduced the management of exudates. Finally, the perspective of this area was given. This minireview gives a comprehensive outline for readers and is believed to provide references for the design of superwettable materials in biomedical area.

## Introduction

As the biggest organ in vertebrates, skin functions as the external barrier for the protection of inner organs, regulation of the body temperature etc. (Morgado et al., 2015). Skin tissue suffers from severe damages when high heat, pressure, genetic disorder and other related diseases occur, and might fail in self-regeneration unfortunately (Divyashri et al., 2022). The injured wounds are classified based on the cause, depth, complexity and time of healing (Iacob et al., 2020). Among which, the chronic wound (in comparison with acute wound) usually fails to heal after the treatment for more than 4 weeks, which poses great threat to human health (Sweeney et al., 2012). In consideration of this, proper wound dressings are highly demanded to protect the wound region and accelerate the healing process.

The first wound bandage can be traced back to 4000 years ago, which was made by honey or resin (Farahani and Shafiee, 2021). After thousands of years evolution, a variety of materials have been applied in wound dressing, such as hydrocolloids, films, foam and hydrogel. Ideal wound dressings should meet the requirements of good biocompatibility, moisture retention, appropriate mechanical property, non-adherent and proper exudate management (Liang et al., 2021). It should be noted that the feasible management of biofluid exerts a crucial role in wound care among all characteristics. Regarding to four phases of wound healing that include hemostasis, inflammation, proliferation, and remodeling, the control of bleeding in hemostasis stage is the primary task, followed by the management of exudates in the inflammatory and proliferative phases (Bernardes et al., 2021; Maleki et al., 2021). Although large efforts have been devoted, there still exist disturbing dilemmas for traditional dressings. For example, materials capable of favorable absorption property usually lead to the ingrowth of clotting and new granulation tissues, which might cause secondary injuries for the routine removal.

**FIGURE 1:**
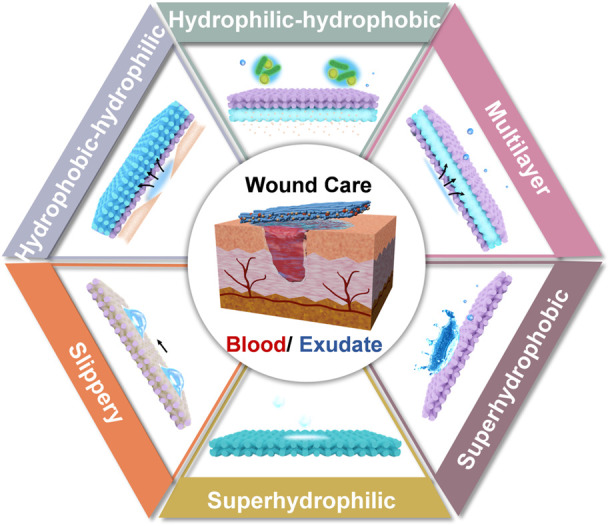
Schematic illustration of superwettable bio-interfaces in wound management.

The development of superwettable materials opens new avenues for wound dressings. Derived from the natural superwettable phenomena, that include the typical examples of self-cleaning lotus leaf and slippery nepenthes, diverse types of superwetability have been explored: superhyrophilic, amphiphobic and Janus wettability (Ding et al., 2012; Zhu et al., 2017; Sun et al., 2021). Depending on this, the exquisite design of bio-interface can address the problems of existing dilemmas in wound dressing. However, the advancements in this area have rarely been combed. In light of this, the aim of this minireview is to summarize the recent strategies of superwettable bio-interfaces for wound care. Concentrating on the management of biofluids, we divided the content into the control of blood (hemostasis) and exudates. The advantages and disadvantages of each design were interpreted, such as superhydrophilic, superhydrophobic, slippery, inner hydrophilic-outer hydrophobic, inner hydrophobic-outer hydrophilic and multilayer materials (Figure 1). Finally, the perspective of this area was given. We have to state that common hydrogels with the intrinsic hydrophilic characteristic lack the feasible tailoring of wettability, and benefit the wound healing from other viewpoints (injectable, self-healing and smart response). This topic is beyond the scope of this review and will not be discussed here. The readers please refer to previous excellent reviews (Dong and Guo, 2021; Liang et al., 2021; Maleki et al., 2021). We believe this minireview will inspire the design of superwettable interfaces, and expand the application of these materials in other related areas.

## Superwettable hemostatic bio-interfaces

The uncontrollable bleeding caused by severe trauma threatens the life of people, haemorrhage control is thus the principal step for wound management (Liu et al., 2022). In an attempt to reduce the high mortality of massive hemorrhage, the development of quick hemostatic materials is in high demand. Normally, the bleeding stops by the formation of blood clot with the main composition of platelets and fibrin (Versteeg et al., 2013). Hydrophilic materials, cotton gauze, as an example, possessing the advantage of high water affinity can absorb water in the blood and accelerate clotting (Zhu et al., 2018). By virtue of this, active hemostatic agents including proteins, polysaccharides and silicon-based materials were widely integrated in hydrophilic substrates for synergistic hemostasis by activating the coagulation cascade, which can be fabricated in the form of membranes, sponges, hydrogels and particles. This topic has been well-summarized by Prof. Guo and will not be discussed here (Liang et al., 2021).

Although large advancements have been achieved, normal hemostatic interfaces constructed by hydrophilic materials generally suffer from the following limitations: 1) massive blood loss due to the rapid capillary drainage of hydrophilic materials; 2) secondary injuries caused by the removal of wound dressing, considering the clotting has formed between bleeding tissue and materials. The delicate design of superwettable bio-interfaces provides an intriguing solution to tackle these problems.

**FIGURE 2:**
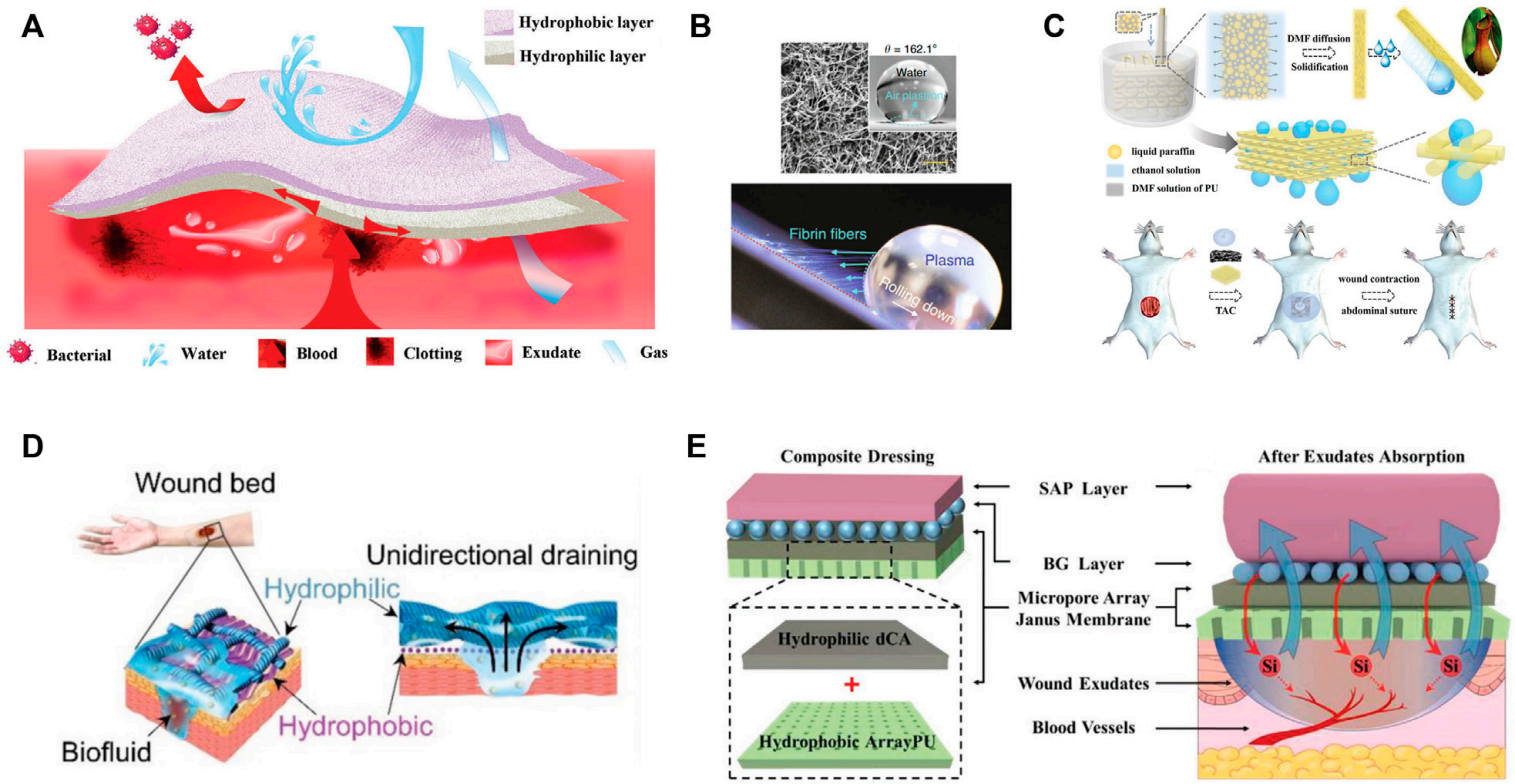
**(A)** Schematic illustration of Janus sponges for wound repair. **(B)** Super-hydrophobic surface with immobilized carbon nanofibers for hemostasis. SEM image and water contact angle of the surface (upper), long fibrin fibers generated when platelet poor plasma droplet rolled down (bottom). **(C)** Schematic illustration of the fabrication process of the 3D-structured slippery polyurethane textile and application in vacuum sealing drainage therapy. **(D)** The design of self-pumping dressing by exudate drainage. **(E)** Scheme of four-layer wound dressing with properties of self-pumping and ion backflow.

Regarding to the massive blood loss, asymmetric or Janus dressings with superhydrophilic and superhydrophobic properties on two sides were adopted. For example, cotton fabric was endowed with Janus performance by spraying one side with hydrophobic SiO_2_ nano-particles and ethyl-α-cyanoacrylate superglue, meanwhile the other side of fabric remained superhydrophilic (Sasaki et al., 2016). The superhydrophilic surface absorbed blood and expedited clotting while the superhydrophobic side prevented the permeation of water and blood. Zhu et al. reported that the Janus fabrics could reduce blood loss more than 50% in comparison with the common superhydrophilic gauzes and prolong the survival time in rat model (Zhu et al., 2018). The similar results were duplicated on Janus cellulose sponges (Cheng et al., 2020). It was explained that the hydrophobic layer prevented the blood penetration and posed proper pressure on the wound, which accounted for efficient blood clotting performance (Figure 2A). This strategy was further verified in self-assembled dipeptide aerogels (Li et al., 2020) and carboxymethyl chitosan/paraffin modified cellulose films (Wang et al., 2020). Moreover, the superhydrophobic external layer, acts as an armor and protects the inner wound from bacterial infection.

To avoid the secondary injuries, the affinity between bleeding tissue and materials should be weakened, and hydrophobic blood-repelling material seems an optimal choice (Liu et al., 2020). However, it should be noted that mere blood repellence fails in the control of massive bleeding. In this case, the active hemostatic agents were simultaneously incorporated. Li and co-workers creatively proposed an approach to address the dilemma of rapid blood coagulation and facial wound-dressing removal (Li Z. et al., 2019). Specifically, a super-hydrophobic surface with immobilized carbon nanofibers (CNFs) was developed, among which CNFs promoted quick fibrin growth and rapid clotting, while the air pocked generated minimal contact area and easy detachment of mature clots (Figure 2B). The peeling tension was 1-2 folds lower than the commercial products. Besides, the commercial zeolite gauze modified with a paraffin coating was rendered with blood repellent property and procoagulant performance as well, which was derived from the retained cation exchange capacity of zeolites (Zhang W. et al., 2021). Very recently, He et al. tailored the hydrophilicity-hydrophobicity balance of cotton gauze by modifying it with catechol compound with flexible long hydrophobic alkyl chain (He H. et al., 2022). The adhesion/blocking effect of catechol groups, blood wicking of cotton and hydrophobic effect of alkyl chains conjointly contributed to the outstanding hemostatic efficiency (rapid hemostatic, low blood loss and no secondary injuries). Following the similar idea, superhydrophilic/superhydrophobic alternate pattern was designed to realize the balance between hemostatic and reduced adhesion (Long et al., 2021).

## Superwettable bio-interfaces for exudate management

Apart from the haemorrhage control in the first step of wound care, the management of exudate is also crucial to wound healing (Sweeney et al., 2012). Wound exudate is defined as the fluid produced around wound in the inflammatory and proliferative phases when haemostasis has been completed (Bernardes et al., 2021). For the acute wounds, the exudate is mainly composed of water electrolytes, protein-digesting enzymes, macrophages, inflammatory mediators and neutrophils, which allows the autolysis of damaged tissue, and provides nutrient for the metabolism of cells (Spear, 2012). The exudate is regarded positive in acute wounds and its production declines with the passage of time. In this case, the dressings should perform the protection and moist control roles (Priya et al., 2016; Shi et al., 2020). By contrast, the composition of chronic wound exudate shows distinct differences: it contains higher concentrations of pro-inflammatory cytokines, matrix metalloproteinases (MMP), and decreased levels of growth factors (Brett, 2006). In addition, the exudate damages peri-wound skin and its production in chronic wounds might be continuous and excessive due to inflammation (Cutting, 2003). Moreover, high-volume exudate could raise the risk of bacterial invasion because the saturated dressing functions as a portal. In this sense, it is of great significance to remove excessive exudate while maintain a moist environment for wound healing.

Water absorbent dressings made by hydrophilic materials supply abundant channels for exudate drainage and are great candidates for exudate management (Varaprasad et al., 2020). In general, the hydrophilic dressings were endowed with other functions including antibacterial and electro-activity (Mayandi et al., 2020; Li et al., 2021; Wang et al., 2022). For instance, superhydrophilic electrospun gelatin nanofibers were loaded with ε-polylysine (a broad-spectrum antibacterial agent) and crosslinked by polydopamine (PDA) for the treatment of second-degree burns (Mayandi et al., 2020). The gelatin mats absorbed the exudates and propelled the migration of early bacterial colonizers into the antibacterial trappers. Li et al. reported nanozyme composite cryogels with the capability of exudate absorption and acid triggered sterilization (Li et al., 2021). The macroporous structure and excellent hydrophilicity of cyogel generated rapid liquid absorption property. Furthermore, taking advantage of the pH-responsive amine groups and switchable Schiff base reaction, on the one hand, the polymer scaffold and Fe-MIL-88NH_2_ nanozyme were positively charged to capture microbes through electrostatic interaction in acidic condition. On the other hand, the nanozyme conducted reversible release and rebinding behavior for dynamic killing of microbes. Wang and coworkers opened a new path for the utilization of exudate by transmitting endogenous bioelectricity and motivating the cascade release of growth factors (Wang et al., 2022). To achieve this goal, short nanofibrous sponges were modified by graphene oxide, which was then reduced to conductive graphene under the effect of PDA, and VEGF-carrying liposomes were then loaded. The above works represent the characteristic examples of hydrophilic bio-interfaces, which regulated the biofluid by simple absorption. However, the hygroscopic dressing might overhydrate the wounds and complicate the healing (Schuren et al., 2005). Besides, new granulation tissues grown in the hydrophilic mats further hinder the routine change of dressing. Therefore, other strategies based on superwettbility interfaces have been proposed.

### Hydrophobic/slippery surfaces

By contrast with the hydrophilic surfaces, superhydrophobic or slippery interfaces are resistance to biofluid, bacteria and cells, which is also known as great anti-fouling property (Zhang H. et al., 2022). These merits perfectly cope with the adhesion of bacteria and new grown tissue, and have exerted great application in wound management. Zhang and coworkers pioneered the utilization of slippery material by fabricating microfiber textiles with liquid-infused porous surface (Zhang et al., 2020). Benefiting from the super low adhesion of biofluid and cells on surfaces, the wound exudation efficiency could be significantly enhanced with neglectable tissue injury in a vacuum sealing drainage therapy (Figure 2C). This anti-adhesion surface can also be integrated in functional patch to prevent the adhesion of wounded intestine with surrounding tissue (Li et al., 2022), and endowed with bactericidal characteristic by the introduction of antibacterial silver nanoparticles (Shi G. et al., 2019).

Aside from the nepenthes-inspired slippery surface, hydrophobic surface combining the advantage of hydrophilic interface was skillfully constructed by microfluidic-emulsion-templating method (Yao et al., 2020). Porous polyvinyl alcohol hydrogel membrane loaded with zeolitic imidazolate framework-8 not only exhibited good repellence to blood and body fluids, but also enabled the controlled release of zinc irons for the eradication of bacterial and the promotion of angiogenesis and collagen deposition.

### Janus materials

Natural human skin is asymmetric with epidermis and dermis arranged from the outer to the inner side (Wu et al., 2016). The epidermis is dense and hydrophobic to resist the bacterial adhesion and avoid excessive dehydration, while the inner dermal layer is sponge-like and hydrophilic to support nutrient exchange and facilitate metabolism. Inspired by this, extensive efforts have been devoted to fabricate asymmetric dressing from the viewpoint of skin tissue engineering. In analogy to natural skin structure, the hydrophilic surfaces resembling that of dermis were normally taken as the inner side for direct interaction with tissue. In order to render the surface with regenerative property, the topological morphology of materials could be engineered to facilitate cell behavior. Besides, growth factors and therapeutic agents were often loaded to augment the effect. In the meanwhile, the hydrophobic surfaces mimicking epidermis were adopted as the external protective layer to defend against bacterial attack.

As for the bioactive side, fibrous membranes and hydrogels provide supportive environments for drug loading and surface structuration. While the protective side can be constructed by various types of hydrophobic materials. As examples, hydrophilic core/shell fibrous membrane was prepared by coaxial electrospinning with curcumin and antimicrobial peptides loaded in the core and shell respectively. Subsequently, poly (lactic acid) (PLA) beads were electro-sprayed on one side of the membrane for superhydrophobic surface (Li W. et al., 2019). The release of antimicrobial peptides and curcumin favored for the treatment of the acute inflammatory response and mid to late wound healing stages correspondingly. And the superhydrophobic surfaces prohibited the adhesion and invasion of exogenous bacteria. Analogously, pioglitazone-incorporated gelatin and poly (ε-caprolactone) (PCL) were electron-spined on two sides of nylon mesh separately (Yu et al., 2020). This asymmetric composite dressing promoted the healing of full-thickness skin wound in diabetic mice (both type 1 and 2 diabetes). You and coworkers fabricated a Janus patch with resveratrol-loaded hydrogel and hydrophobic polymer (You et al., 2015). Taking advantage of the hydrophobic blocking polymer, the fluid penetration, accompanied with the diffusion of resveratrol was monodirectional, which was beneficial for efficient drug delivery. The hydrogel layer of functional Janus membrane could also be loaded with growth factors and the hydrophobic surface was reported to prevent exudate leaks (An et al., 2017). Interestingly, anisotropic microgrooved hydrogel was designed to facilitate cell behavior (adhesion, proliferation, and oriented migration), and the other side was endowed with anti-adhesive performance by the infusion of liquid paraffin (Zou et al., 2020). This Janus patch avoided adhesion-related complications and promoted the repair of abdominal wall defects in rat. Moreover, superwettable asymmetric polyurethane (PU) sponges were successfully prepared by spraying fluorinated zinc oxide nanoparticles onto one surface of PDA-modified sponge (Chen et al., 2022). It was demonstrated that the durable bacterial barrier of superhydrophobic coatings was conductive to the healing of infected wound.

In contrast to the inner hydrophilic-outer hydrophobic strategy, inner hydrophobic-outer hydrophilic design features the unidirectional transport of exudates and feasible change of wound dressings. This concept was proposed by Shi and coworkers for the first time (Figure 2D), who elaborated the mechanism and key points for the design of self-pumping dressing (Shi L. et al., 2019). The density of hydrophobic nanofiber arrays and the multiple contacting points on hydrophilic microfibers contributed to the rapid transport of biofluids to the hydrophilic side once they were in contact with the hydrophobic fibers. More importantly, the intrinsic hydrophobicity of fabrics resisted the ingrowth of regenerated tissue. Since then, a variety of bio-interfaces including polymeric fibers (Luo et al., 2021), polyurethane sponges (Zhang H. et al., 2021), porous films (Chi et al., 2021), hydrogel composite membrane (Zhang J. et al., 2022) and cotton fabrics (Zhang Z. et al., 2022) have been well-developed. To the best of our knowledge, the absorption and retainment of biofluid is closely associated with the intrinsic property of hydrophilic substrates. In regard to this, the reverse penetration of liquid from the hydrophilic substrate might occur if the water retention capability is not satisfactory. To address this problem, nanofibrous composite aerogel served as the hydrophilic absorption layer to prohibit the reverse liquid penetration (Zhang K. et al., 2021). In brief, quaternized chitosan/polyvinyl alcohol aerogel overcame low water absorption property of traditional compact nanofibers, meanwhile retained the soft mechanical property. After the coverage of hydrophobic curcumin-loaded PCL, the hybrid dressing was utilized for diabetic wound therapy by virtue of antibacterial, antioxidant, and fluid gating characteristics. From another perspective, Qian et al. raised the management of exudate by the collection of wound exudate in the early stage, followed by the cascade release of drug (curcumin) from the hydrophilic substrates (Qian et al., 2022). The inverse effects of the hydrophilic layers in above two works might be attributed to diverse absorption capabilities of hydrophilic substrates and different blocking ability of hydrophobic layer.

### Multilayer designs

Superior to single-layer or double-layer dressings, multilayer substrates are designable for the attainment of more complex functions. He et al. reported a three-layer composite dressing consisted of PCL/gelatin nanofiber, collagen/quaternized chitosan sponge and PCL/polystyrene microspheres from the inner to the outer side (He C. et al., 2022). The aligned PCL/gelatin together with curcumin promoted directed cell growth and migration, sponges absorbed exudates, and hydrophobic microspheres inhibited exogenic bacterial adhesion. This strategy enables the antibacterial role of both inner and outer sides. Obeying the same rule, superhydrophilic-hydrophilic-hydrophobic-superhydrophobic four-layer hybrid substrates were developed with the integration of ciprofloxacin and astaxanthin in the hydrophilic layer (Zhang et al., 2023). The combination of fluid absorption, antibacterial, antioxidation and self-cleaning gave rise to potential applications of this membrane in skin tissue engineering.

For the purpose of full utilization of exudates, Chang’s group designed a four-layer composite dressing for self-pumping and ion backflow (Bao et al., 2020). PU membrane with micropore arrays served as the bottom layer, while hydrophilic deacetylated cellulose acetate, bioactive silicate bioglass particles and superabsorbent particles were placed above in sequence (Figure 2E). This system allows for unidirectional transport of abundant exudates from wound bed, and simultaneous backflow of a small amount of fluid (containing bioactive ions). This novel bi-functional transport process benefited the regeneration of diabetic wound. Furthermore, this design can be simplified by sandwich structure: hydrophilic zinc silicate bioceramics encapsuled by hydrophobic PLA in both sides (Zhang Z. et al., 2021). The exudate absorption followed by the release of Zn^2+^ and SiO_3_^2-^ ions facilitated the healing of burn wound and regeneration of appendage (hair follicle).

To surmount the low stretching capability of traditional band aids, Xu and coworkers intelligently fabricated a three-layer patch using Janus polydimethylsiloxane layer, filter paper and medical adhesive tape (Xu et al., 2021). Polydimethylsiloxane layer was drilled by femtosecond laser and then single-side modified for Janus property. This patch possessed unidirectional liquid transport on both relaxed and stretched states, indicating the application on moveable skin wounds (stretched or bended skin surface).

## Conclusion

In summary, the development of superwettable bio-interfaces motivates the application of these designs in wound care, which exhibit attractive advantages of biofluid management, drug delivery, easy to change etc. In view of the two primary biofluids during healing stages (blood and exudates), superwettable hemostatic bio-interfaces were first described, the management of exudates was then summarized. The blood management requires the quick hemostatic, no blood loss and secondary injuries, and the integration of superhydrophobic bio-interface satisfies the needs. Diverse strategies including hydrophobic/slippery, inner hydrophilic-outer hydrophobic, inner hydrophobic-outer hydrophilic and multilayer surfaces were applied for exudate management. These bio-interfaces all have distinctive merits and were reasonably verified in the specific wound model. Based on the shallow understanding of authors, the perspectives were proposed as follows: 1) The delicate control of exudates (the backflow and purification) needs in-deep investigation, especially the mechanism and accuracy. 2) Almost all chronic animal models in the reported papers differ from that in clinic (non-healing for more than 4 weeks), and the animal model resembling that of real case would be more meaningful. 3) The regeneration of skin appendices and scarless wound healing are still challenging. In a word, tailoring superwettability bio-interfaces provides inspiring solutions to tackle the existing problems in wound management. We hope that this minireview can give a comprehensive outline for readers and provide references for the design of superwettable materials in biomedical area.
